# Prevalence and risk factors of postpartum depression among women living in the United Arab Emirates

**DOI:** 10.1007/s00127-022-02372-1

**Published:** 2022-10-14

**Authors:** Nivine Hanach, Hadia Radwan, Randa Fakhry, Cindy-Lee Dennis, Wegdan Bani issa, MoezAlIslam E. Faris, Reyad Shaker Obaid, Suad Al Marzooqi, Charbel Tabet, Nanne De Vries

**Affiliations:** 1 Faculty of Health, Medicine, and Life Sciences, CAPHRI, Maastricht University, P.O. Box 616, Maastricht, Netherlands; 2 Department of Clinical Nutrition and Dietetics, College of Health Sciences, Research Institute of Medical and Health Sciences (RIMHS), University of Sharjah, Sharjah, United Arab Emirates; 3 Department of Nursing, College of Health Sciences, Research Institute of Medical and Health Sciences (RIMHS), University of Sharjah, Sharjah, United Arab Emirates; 4 Lawrence S. Bloomberg, Faculty of Nursing, University of Toronto, Toronto, Canada; 5 Department of Psychology, Al Ain, United Arab Emirates University, Al Ain, United Arab Emirates; 6 CREG, Grenoble Alpes University, Paris, France

**Keywords:** Postpartum period, Postpartum depression, Prevalence, Risk factors, Maternal mental health

## Abstract

**Purpose:**

Postpartum depression received almost no attention in the United Arab Emirates (UAE). The aim was to examine the prevalence of depressive symptomatology and the associated risk factors among women in the UAE.

**Methods:**

A prospective cohort study recruited women from postpartum wards in hospitals across four emirates in the UAE. Women completed questionnaires immediately after childbirth and at 3 and 6 months postpartum. Depressive symptomatology was measured using the Edinburgh Postnatal Depression Scale (EPDS > 12). Risk factors were identified using the generalized estimating equation. A stratified analysis of the postpartum period was performed.

**Results:**

Among the 457 women recruited, 35% exhibited depressive symptomatology within the first 6 months postpartum. Younger women (< 25 years), part-time employment, the receipt of financial support from the family, and difficulty in managing monthly income were associated with a higher risk of postpartum depression. Husband’s employment, husband’s support, and living in own house were associated with a lower risk of postpartum depression. Maternity leave of more than 3 months increased the risk of depression during the first 3 months postpartum. From 3 to 6 months postpartum, Muslim women had a higher risk of depression whereas women who breastfed other children and in the past 7 days, and perceived their infant as healthy had a lower risk of depression.

**Conclusions:**

The prevalence of maternal depressive symptomatology is considerable in the UAE. Risk factors change over the 6-month postpartum period suggesting the need for an innovative multidisciplinary approach to the management of postpartum depression, including follow-up screening.

## Introduction

Postpartum depression is a serious mood disorder that affects more than one in nine women worldwide [1] and often continues past the first year postpartum if unrecognized [2]. Globally, the overall prevalence of postpartum depression among women is suggested to average 17% [3] but ranges from 4.0 to 63.9% [4]. In the Arab region, Ayoub, Shaheen, and Hajat (2020) recently reported in a systematic review of 25 studies that the prevalence of postpartum depression ranged from 15 to 25% in 12 studies, less than 15% (with a minimum rate of 7%) in 7 studies, and more than 25% in 6 studies (with a maximum 74%). It is characterized by a persistent feeling of sadness and low mood, loss of interest in activities, sleep disturbances, appetite changes, negative and guilty thoughts, difficulty in concentrating and making decisions, lack of energy and feeling utterly tired, excessive irritability or anger, and difficulty bonding with the baby [6]. It is frequently undiagnosed [7] resulting in a broad array of adverse effects on the mother, infant, and the family. It could cause psychiatric hospitalization, affect the child’s cognitive, social, and behavioral functioning, as well as impact the family dynamics [8].

The postpartum period is a transitional phase during which the mother undergoes multiple physical, social, and psychological changes. This includes fatigue, weight loss, financial strain, decreased partner support, and childcare stress [9]. Given these additional concerns, it is important to identify culturally specific risk factors of postpartum depression to enable effective prevention strategies that facilitate the transition to motherhood. While the etiology of postpartum depression is multifactorial, international studies have consistently found low socioeconomic status, decreased levels of perceived social support, poor partner relationship, increased number of stressful life events, and history of depression to be significant predictors among women [5, 8, 10].

In the United Arab Emirates (UAE), there is a substantial discrepancy in the reported prevalence of postpartum depression with rates varying widely from 12 to 33% [7, 11, 12]. This could be attributed to the country’s ongoing rapid growth and the distinctive cultural diversity within the same settings: over 80% of the population is of non-UAE nationals with more than 200 different nationalities [13]. Evidence suggests that maternal employment status, age, number of children, relationship with partner, and infant-feeding practices are significant predictors influencing maternal postpartum mood among women in the UAE [7, 11, 12]. However, the status of postpartum depression in the UAE remains understudied and no previous studies have been conducted across different emirates of the UAE where women from different nationalities were recruited—which represents the actual UAE population. Furthermore, with the numerous potential risk factors, it is vital to establish an understanding of their impact on depression, particularly during the postpartum period, to assist in the development of culturally specific preventive interventions. Therefore, the present study has been undertaken to (1) determine the prevalence rates of postpartum depressive symptoms among multi-ethnic and multi-cultural women living in the UAE and (2) investigate the associated factors including sociodemographic, obstetric, and social support variables over 6 months postpartum. Our secondary goal is to demonstrate whether the impact of risk factors on depressive symptomatology varied from the early (immediately after childbirth to 3 months) to the late (3–6 months) postpartum period. This consequently aims to enhance targeted mental health support and services for postpartum women in the UAE.

## Methods

### Study design

This study is part of a larger multicenter, prospective cohort study conducted in the UAE [14]. The study was approved by the research ethics committees at the University of Sharjah (REC/16-04-14), the Ministry of Health and Prevention (MOHAP/DXB/SUBC/No.37/2017), and Dubai Health Authority (DSREC-11/2017_01).

### Study setting and population

The study was conducted over 6 months across four emirates of the UAE (Sharjah, Dubai, Abu Dhabi, and Fujairah) in seven approved public and private hospitals. Using convenience sampling, a team of trained research assistants (RAs) recruited participants from the maternity wards. Women were eligible if they met the following inclusion criteria: (1) literate Emirati or expatriate, (2) 1–2 days postpartum, (3) between 18–45 years old, and (4) had a singleton birth. Women were excluded if they had a preterm birth (before 37 weeks gestational age) or an infant in the NICU who would not be discharged home with the mother. The RAs informed the eligible participants about the aim and objectives of the study and their intended involvement process prior to providing written informed consent. Participation was completely voluntary and participants were assured confidentiality of all the collected information. The enrolled mothers completed questionnaires with RAs at three time-points: face-to-face immediately after childbirth (T1) and via telephone at 3 months (T2) and 6 months (T3) postpartum. They received a gift voucher upon completion of each study questionnaire. Data collection was completed between February 2018 and July 2019.

### Sample size

We conducted a priori calculation of the sample size using Power Analysis and Sample Size System version 11 (NCSS software, Utah, USA). With a 33% prevalence of postpartum depression previously reported in the UAE [7], the available sample size (*N *= 374) allowed the estimation of a prevalence of 35% at a power of 80% and an error of 5%.

### Data collection

At T1, the RAs collected sociodemographic information of the participants and their spouses in addition to obstetric data including breastfeeding practices, complications during pregnancy, labor, postpartum care, living arrangements, social support (from husband, parents, and in-laws), and childcare assistance (from a nanny and or house helper). Infant data [birth weight (g), length (cm), and Apgar score (at 1 and 5 min)] were also reported from the hospital medical records. At T2 and T3, women were contacted by the same RAs to collect information on the maternal and infant health including anthropometrics [maternal body mass index (BMI) = kg/m^2^ − calculated and evaluated according to the World Health Organization classification and infant weight (g) and length (cm)], breastfeeding practices, living arrangements, work status, marital status, social support, and childcare assistance. At the three follow-up points, the participants were asked to fill out the Edinburgh Postnatal Depression Scale (EPDS). To account for language differences among the participants, the RAs had to be fluent in Arabic and English and questionnaires were pilot tested in both languages prior to use. Variables were classified into two categories: fixed and time-dependent. Fixed variables are those where the effects do not change or change at a constant rate over time and include background demographics and all the obstetric data that were collected immediately after childbirth. Variables that are predicted to change over time were considered time-dependent and included maternal work status, marital status, BMI, breastfeeding in the past 7 days, perception of infant health, paternal support in childcare, and current living arrangement.

### Instruments

*Edinburgh Postnatal Depression Scale* (EPDS), a 10-item self-report scale was used to identify women with depressive symptomatology [15]. The total score ranges from 0 to 30, with each item being scored on a four-point scale from 0 to 3. Higher scores indicated increased symptomatology. In the present study, we used the following cut-off points: EPDS < 10: no depression; EPDS 10–12: minor depressive symptomatology; EPDS > 12: high depressive symptomatology. Women with high depressive symptomatology were referred to a clinical psychologist. EPDS is systematically demonstrated to be a valid screening tool for depression among perinatal women [16] Furthermore, a validated Arabic version with adequate psychometric properties [17] as used with Arabic-speaking participants. The Cronbach’s alpha of EPDS in this study was 0.795 at T1, 0.854 at T2, and 0.861 at T3.

### Statistical analysis

Collected data were analyzed using the Statistical Package for the Social Sciences software version 26.0 (IBM Corp, New York, USA). Descriptive statistics (frequency count and percentages) were used to summarize sample characteristics. A chi-squared test was conducted to compare dichotomous sociodemographic, obstetric, and social support variables based on EPDS scores, immediately after childbirth. To account for the longitudinal nature of our data we used a generalized estimating equations (GEE) analysis to explore significant risk factors associated with postpartum depression symptomatology across time. An exchangeable correlation structure and ordinal logistic link function were specified. With the aim to examine significant risk factors of postpartum depression over the 6-month postpartum period, we first conducted a cumulative GEE analysis (Model 1; T1–T3) where all variables assessed at the three time-points were included. To address the objective of determining variables associated with postpartum depression specifically in the early versus late postpartum periods, two separate GEE models were further performed. Model 2 (from 0 to 3 months) included variables assessed at T1 and T2 and Model 3 (from 3 to 6 months) included variables assessed at T2 and T3. The selection of the variables in the models was based on the existing empirical clinical evidence. Model 1 and 2 included all sociodemographic, obstetric, social variables, and infant characteristics. Model 3 included the same parameters as model 1 and 2 in addition to feeding practices variables. The results of the estimates are presented as adjusted odds ratios (OR) with the corresponding two-sided 95% confidence intervals and *P* values. In this study, point prevalence was calculated by dividing the proportion of women with EPDS > 12 at T1, T2, and T3 by the corresponding total number of women (*n*) at these particular time points. Period prevalence was calculated by dividing the total number of women who reported an EPDS > 12 over the 6-month period by the total sample size (N) [18]. A one-way repeated measures analysis of variance (ANOVA) was performed to compare the mean of EPDS score across time. A *p* value of < 0.05 is considered statistically significant.

## Results

### Sample characteristics

A total of 457 women participated in the study of which 399 (87.3%) completed the 3-month questionnaire and 374 (81.2%) completed the 6-month questionnaire. The mean age of the participants was 31.38 ± 5.53 years old and the majority (*n* = 328, 71.7%) were of Arab nationality and reported Islam as their religious affiliation (*n* = 367, 80%). Over half of the women (*n* = 295, 64.5%) had a university degree and 61% (*n* = 278) were not working. Among those who were working (*n* = 179), 86% worked on a full-time basis (*n* = 155) and had maternity leave for more than 3 months (*n* = 154). A total family income of 10,000 AED or more per month was reported by 58.8% (*n* = 269). Regarding maternal and infant characteristics, 34.7% (*n* = 159) of women reported having experienced complications during pregnancy and the majority were multiparous (*n* = 353, 77.2%), had a vaginal delivery (*n* = 270, 59%), and were either overweight or obese (*n* = 229, 50.1%). Only 70% of newborns were breastfed within the first hour of birth (*n* = 320), 51% were males (*n* = 233), and 88.4% (*n* = 404) weighed between 2500 and 4000 g (mean 3.16 ± 1.48 kg). All newborns had normal 1-min (mean 8.47 ± 0.89) and 5-min (mean 9.36 ± 0.59) Apgar scores between 7 and 10.

### Prevalence of postpartum depression

**Fig. 1:**
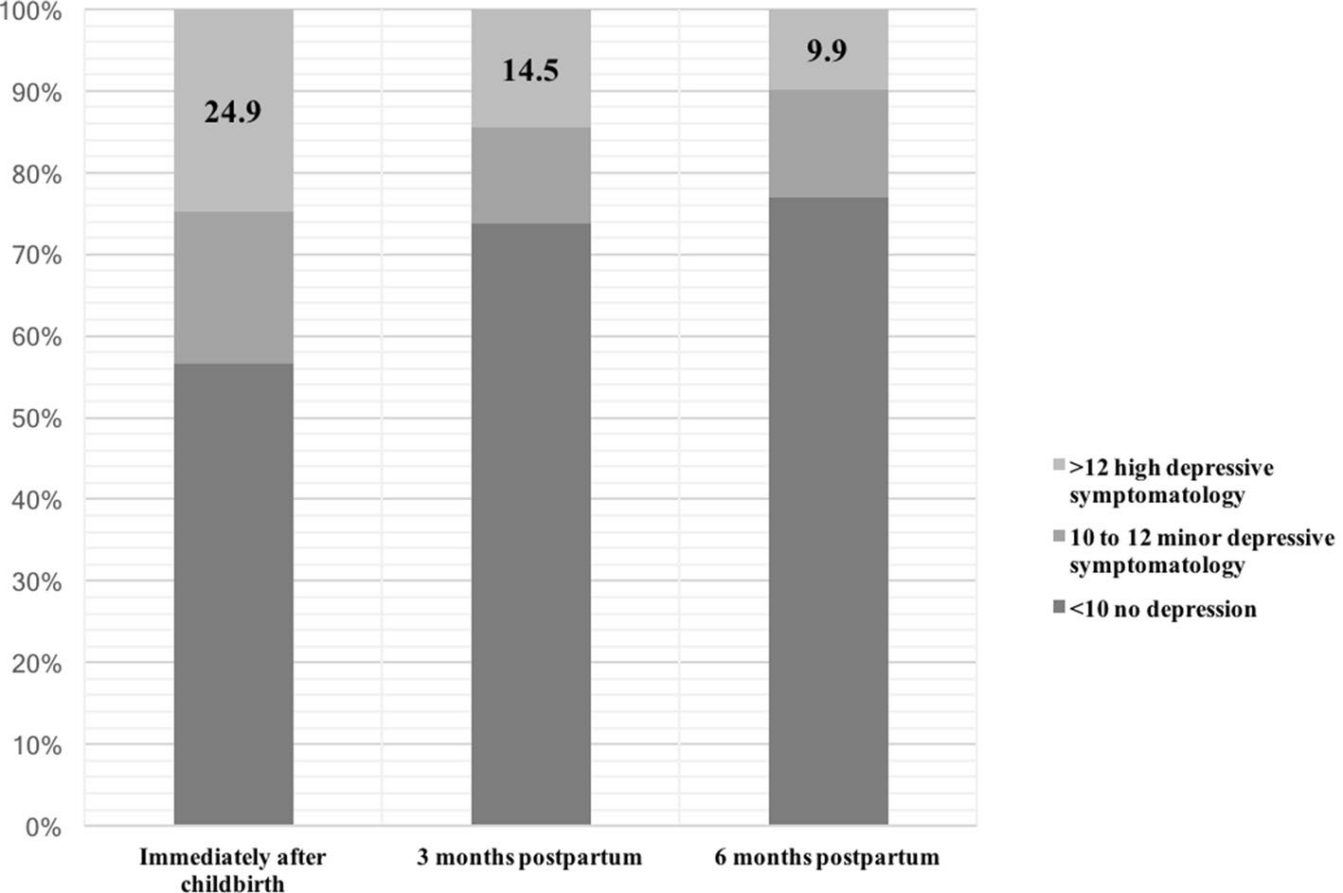
Point prevalence of postpartum depression among women living in the UAE, immediately after childbirth, at 3- and 6-month postpartum

The overall prevalence postpartum depression symptomatology (EPDS > 12) over the 6-month period was 35% (*n* = 160/457). Figure 1 illustrates the distribution of EPDS scores at each time point. Immediately after childbirth (T1) 24.9% (*n* = 114/457) of women had an EPDS > 12 decreasing to 14.5% (*n* = 58/399) at 3 months postpartum (T2) and 9.9% (*n* = 37/374) at 6 months postpartum (T3). Repeated measures ANOVA showed a significant interaction between time and the mean EPDS scores [*F*(2, 746) = 58, *p* < 0.001]. While mean EPDS scores changed significantly from T1 to T2 (mean difference: − 2.39; SE = 0.32; *p* < 0.001) and T1 to T3 (mean difference: − 2.97; SE = 0.3; *p* < 0.001) no difference was detected between T2 and T3 (mean difference: − 0.58; SE = 0.24; *p* = *0*.057).

### Distribution of maternal characteristics by EPDS score

Table 1 compares the distribution of demographic, obstetric, and social variables by EPDS scores among women at T1. EPDS scores were significantly different between women in terms of nationality, religion, ability to manage monthly family income, the receipt of financial support from the family, and parity. Nearly 28% of women who were of Arab origin (*p* = 0.02) and Islam faith (*p* = 0.01) had an EPDS > 12 in comparison to those of other nationalities and religions. Women who reported that managing their monthly family income was difficult sometimes (32.3%) or all the time (81.3%) had higher EPDS scores compared to those who found it easy (15.9%) or not bad (23%) (*p* < 0.001). Of those who received financial support from their family, 38.4% had an EPDS > 12 compared to 19.2% of those who did not (*p* < 0.001). Interestingly, the maternal EPDS scores were also significantly associated with the husband’s characteristics including nationality, employment status, and educational level. Women whose husband was of Arab origin (28.7%; *p* = 0.03), were not employed (58.3%; *p* < 0.001), and only received a high school diploma or less (*p* < 0.001), reported a high EPDS level > 12. None of the obstetric variables was statistically significantly associated with EPDS scores except for parity were significantly more multiparous than primiparous women had an EPDS > 12 (27.5% versus 16.5%, respectively; *p* = 0.04). Husband support was also statistically significantly associated with lower EPDS scores, 55.6% of women who did not receive husband support immediately after childbirth had an EPDS > 12 compared to 24.3% of those who received husband support (*p* = 0.02).

**Table 1:** Comparison of maternal baseline characteristics and EPDS scores immediately after childbirth

Variable	*N*	Maternal EPDS scores (*N* = 457)				
		EPDS < 10	EPDS 10–12	EPDS > 12	*X*^*2*^ (*df*)	*P* value
		*n* (%)	*n* (%)	*n* (%)		
Sociodemographic						
Mother's age						
18–24.9	54	31 (57.4)	7 (13)	16 (29.6)	7.8 (4)	0.099
25–29.9	112	52 (46.4)	27 (24.1)	33 (29.5)		
≥ 30	292	175 (60.1)	51 (17.5)	65 (22.3)		
Mother’s nationality						
Arab/Emirati	328	173 (52.7)	62 (18.9)	93 (28.4)	10.8 (4)	0.029
Asian	104	65 (62.5)	20 (19.2)	19 (18.3)		
Western/other	25	20 (80)	3 (12)	2 (8)		
Mother’s religion						
Muslim	367	194 (52.9)	70 (19.1)	103 (28.1)	12.5 (4)	0.014
Christian	68	50 (73.5)	11 (16.2)	7 (10.3)		
Hindu/Buddhism	22	14 (63.6)	4 (18.2)	4 (18.2)		
Mother’s education level						
Less than high school	29	10 (34.5)	6 (20.7)	13 (44.8)		
High school	105	55 (52.4)	22 (21)	28 (26.7)	11.1 (6)	0.084
Technical diploma	30	21 (70)	5 (16.7)	4 (13.3)		
University degree	293	172 (58.7)	52 (17.7)	69 (23.5)		
Work status						
Full-time	154	93 (60.4)	30 (19.5)	31 (20.1)	5.2 (4)	0.265
Part-time	24	11 (44)	4 (16)	10 (40)		
Not working	278	154 (55.4)	51 (18.3)	73 (26.3)		
Maternity leave						
< 3 months/no maternity leave	19	10 (52.6)	3 (15.8)	6 (31.6)	1.51 (4)	0.824
≥ 3 months	155	92 (59.4)	29 (18.7)	34 (21.9)		
Not working/not returning to work	283	156 (55.1)	53 (18.7)	74 (26.1)		
Marital status						
Married	457	218 (47.7)	125 (27.4)	114 (24.9)	–	–
Separated/widowed/divorced	0	0 (0)	0 (0)	0 (0)		
Husband’s nationality						
Arab/Emirati	328	173 (52.3)	63 (19)	95 (28.7)	10.5 (4)	0.032
Asian	93	61 (72)	16 (17.2)	16 (17.2)		
Western/other	25	18 (72)	5 (20)	2 (8)		
Husband’s employment status						
Employed	433	252 (58.2)	81 (18.7)	100 (23.1)	15.9 (2)	< 0.001
Not employed	24	6 (25)	4 (16.7)	14 (58.3)		
Husband’s educational level						
Less than high school	34	15 (44.1)	6 (17.6)	13 (38.2)	23.6 (6)	< 0.001
High school	64	56 (46.7)	18 (15)	46 (38.3)		
Technical diploma	38	23 (10)	10 (26.3)	5 (13.2)		
University degree	265	164 (61.9)	51 (19.2)	50 (18.9)		
Family income						
< 5000 AED	20	7 (35)	5 (25)	8 (40)	15 (8)	0.059
5000–10,000 AED	70	32 (45.7)	12 (17.1)	26 (37.1)		
10,000–15,000 AED	89	48 (53.9)	20 (22.5)	21 (23.6)		
> 15,000 AED	180	114 (63.3)	32 (17.8)	34 (18.9)		
Don’t know/refuse to answer	98	57 (58.2)	16 (16.3)	25 (25.5)		
Management of family income						
Difficult all the time	16	2 (12.5)	1 (6.3)	13 (81.3)	38.2 (6)	0.001
Difficult sometimes	99	46 (46.5)	21 (21.2)	32 (32.3)		
Not bad	204	120 (58.8)	37 (18.1)	47 (23)		
Easy	138	90 (65.2)	26 (18.8)	22 (15.9)		
Mother’s BMI						
Underweight	23	17 (73.9)	3 (13)	3 (13)	8.1 (6)	0.225
Normal	205	117 (57.1)	32 (15.6)	56 (27.3)		
Overweight	142	82 (57.7)	30 (21.1)	30 (21.1)		
Obese	87	42 (28.3)	20 (23)	25 (28.7)		
Mother's self-health perception						
Excellent	123	74 (60.2)	23 (18.7)	26 (21.1)	1.61 (4)	0.8
Good	326	180 (55.2)	60 (18.4)	86 (26.4)		
Poor	8	4 (50)	2 (25)	2 (25)		
Obstetrics						
Complications during pregnancy						
Yes	159	78 (49.1)	34 (21.4)	47 (29.6)	5.4 (2)	0.06
No	298	180 (60.4)	51 (17.2)	67 (22.5)		
Type of delivery						
Vaginal spontaneous	253	153 (60.5)	41 (16.2)	59 (23.3)	5.13 (4)	0.27
Vaginal assisted	16	10 (62.5)	2 (12.5)	4 (25)		
C-section	188	95 (50.5)	42 (22.3)	51 (27.1)		
Receipt of epidural						
Yes	346	198 (57.2)	64 (18.5)	84 (24.3)	0.41 (2)	0.81
No	111	60 (54.1)	21 (18.9)	30 (27)		
Breastfeeding initiation time						
< 1 h	320	191 (59.7)	55 (17.2)	74 (23.1)	6.7 (4)	0.15
≥ 1 h	115	59 (51.3)	25 (21.7)	31 (27)		
Did not breastfeed	22	8 (36.4)	5 (22.7)	9 (40.9)		
Breastfed other children						
Yes	340	182 (55.3)	65 (19.1)	93 (27.4)	5.2 (2)	0.07
No	117	76 (65)	20 (17.1)	21 (17.9)		
Parity						
Primiparous	104	69 (66.3)	18 (17.3)	17 (16.3)	6.4 (2)	0.04
Multiparous	353	189 (53.5)	67 (19)	97 (27.5)		
Sex of the infant						
Boy	233	128 (54.9)	40 (17.2)	65 (27.9)	2.3 (2)	0.3
Girl	224	130 (58)	45 (20.1)	49 (21.9)		
Infant’s birth weight						
< 2.5 kg	35	21 (60)	6 (17.1)	8 (22.9)	3 (4)	0.55
2.5–4 kg	404	228 (56.4)	73 (18.1)	103 (25.5)		
> 4 kg	18	9 (50)	6 (33.3)	3 (16.7)		
Social support						
Parents support						
Yes	391	218 (55.8)	74 (18.9)	99 (25.3)	0.54 (2)	0.76
No	66	40 (60.6)	11 (16.7)	15 (22.7)		
Parents provide financial support						
Yes	125	56 (44.8)	21 (16.8)	48 (38.4)	16.6 (2)	< 0.001
No	265	161 (60.8)	53 (20)	51 (19.2)		
Parents provide emotional support						
Yes	319	182 (55.3)	53 (19.1)	84 (25.5)	0.09 (2)	0.956
No	61	35 (57.4)	11 (18)	15 (24.6)		
Parents provide childcare assistance						
Yes	273	152 (55.7)	54 (19.8)	67 (24.5)	0.56 (2)	0.753
No	117	65 (55.6)	20 (17.1)	32 (27.4)		
Husband support						
Yes	448	257 (57.4)	82 (18.3)	109 (24.3)	7.86 (2)	0.02
No	9	1 (11.1)	3 (33.3)	5 (55.6)		
In-law’s support						
Yes	402	228 (56.7)	78 (19.4)	96 (23.9)	2.7 (2)	0.257
No/not applicable	55	30 (54.5)	7 (12.7)	18 (32.7)		
Living arrangement						
Own home	336	192 (57.1)	67 (19.9)	77 (22.9)	5.7 (4)	0.222
Mother’s home	82	44 (53.7)	15 (18.3)	23 (28)		
In-law’s home/other	39	22 (56.4)	3 (7.7)	14 (35.9)		
Nanny						
Yes	74	44 (59.5)	15 (20.3)	15 (20.3)	1 (2)	0.592
No	383	214 (55.9)	70 (18.3)	99 (25.8)		
House-helper						
Yes	239	130 (54.4)	50 (20.9)	59 (24.7)	1.8 (2)	0.398
No	218	128 (58.7)	35 (16.1)	55 (25.2)		

### Risk factors of postpartum depression

Table 2 presents the results of the performed GEE analysis. Models 1 and 2 adjusted for the following variables: mother’s nationality, mother’s education level, mother’s health perception, husband’s nationality, husband’s nationality, parents' support, family monthly income, complication(s) during pregnancy, type of delivery, receipt of epidural, infant gender, infant weight, parity, marital status, breastfeeding initiation time, family financial support, family emotional support, family childcare support, in law’s support, house helper, nanny, BMI, and time.

**Table 2:** Generalized estimating equations analysis predicting postpartum depression symptoms among women living in the UAE

Variables	Model 1 (T1–T3)				Model 2 (T1–T2)				Model 3 (T2–T3)			
	OR	95% CI		*P *value	OR	95% CI		*P *value	OR	95% CI		*P *value
Age												
18–24.9	1.95	1.18	3.22	0.009	1.77	1	3.14	0.04	1.17	0.02	1.51	0.11
25–29.9	1.22	0.8	1.88	0.345	1.3	0.85	2.08	0.24	0.46	0.05	3.89	0.48
≥ 30	Ref				Ref			Ref				
Work status												
Full-time	0.87	0.46	1.63	0.67	0.74	0.35	1.56	0.44	0.52	0.26	1.01	0.05
Part-time	1.93	1	3.73	0.04	0.83	0.32	2.16	0.71	2.94	1.29	6.72	0.01
Not working	Ref				Ref				Ref			
Mother’s religion												
Muslim	1.8	0.67	4.84	0.24	2	0.74	5.39	0.16	4.65	1.07	20	0.03
Christian	0.75	0.29	1.95	0.56	0.69	0.27	1.78	0.45	0.85	0.17	4.14	0.84
Hindu/Buddhism									Ref			
Maternity leave												
< 3 months/ No maternity leave	0.76	0.41	1.41	0.39	1.12	0.54	2.33	0.74	0.66	0.33	1.34	0.25
≥ 3 months	2.85	0.95	8.52	0.06	4.81	1.52	15.2	0.007	1.79	0.49	6.5	0.37
Not working/Not returning to work	Ref				Ref				Ref			
Family provides financial support												
Yes	1.67	1.11	2.51	0.013	1.56	1.02	2.38	0.03	1.58	0.9	2.77	0.1
No	Ref				Ref				Ref			
Management of monthly income												
Difficult all the time	4.44	2.04	9.66	< 0.001	4.46	1.97	10.1	< 0.001	1.4	0.37	5.31	0.61
Difficult sometimes	2.11	1.29	3.45	0.003	2.07	1.25	3.45	0.005	1.9	0.93	3.89	0.07
Not bad	1.52	1.01	2.29	0.04	1.46	0.95	2.24	0.08	1.88	1.04	3.39	0.03
Easy	Ref				Ref				Ref			
Husband employed												
Yes	0.3	0.14	0.62	0.001	0.31	0.15	0.64	0.001	0.37	0.12	1.15	0.08
No	Ref				Ref				Ref			
Husband support												
Yes	0.5	0.33	0.75	0.001	0.65	0.38	1	0.1	0.47	0.29	0.76	0.002
No	Ref				Ref				Ref			
Living arrangement												
Own home	0.49	0.29	0.83	0.008	0.51	0.28	0.95	0.03	0.34	0.18	0.65	0.001
Family home	0.61	0.32	1.17	0.14	0.68	0.33	1.39	0.29	0.76	0.19	2.96	0.69
In-law's home/other	Ref				Ref				Ref			
Breastfeeding other children												
Yes	0.55	0.24	1.27	0.16	0.65	0.22	1.92	0.44	0.37	0.14	0.97	0.04
No	Ref				Ref				Ref			
Breastfeeding in the past 7 days												
Yes									0.3	0.16	0.56	< 0.001
No									Ref			
Perception of healthy baby												
Yes									0.17	0.04	0.65	0.01
No									Ref			

Model 1 and 2 adjusted for the following variables: mother’s nationality, mother’s education level, mother’s health perception, husband’s nationality, husband’s nationality, parents' support, family monthly income, complication(s) during pregnancy, type of delivery, receipt of epidural, infant gender, infant weight, parity, marital status, breastfeeding initiation time, family financial support, family emotional support, family childcare support, in law’s support, house helper, nanny, BMI, and time

Model 3 adjusted for the same parameters as model 1 and 2 plus the following variables: history of breastfeeding other children, breastfeeding in the past 7 days

*OR* odds ratio, *CI* confidence interval

Model 3 adjusted for the same parameters as models 1 and 2 plus the following variables: history of breastfeeding other children, breastfeeding in the past 7 days. Over the 6-month postpartum period (model 1), the risk of having an EPDS > 12 was significantly higher among women who were less than 25 years old (OR = 1.95, 95% CI 1.18–3.22; *p* = 0.009) and those who worked on a part-time basis (OR = 1.93, 95% CI 1–3.73; *p* = 0.04). Furthermore, women who received financial support from their families (OR = 1.67, 95% CI 1.11–2.51; *p* = 0.013) and had difficulty in managing their monthly income (OR = 4.44, 95% CI 2.04–9.66; *p* < 0.001) were more likely to have depressive symptomatology. The odds ratio for EPDS > 12 was significantly lower for women who felt supported mostly by their husband (OR = 0.5, 95% CI 0.33–0.75; *p* = 0.001), their husband was employed (OR = 0.3, 95% CI 0.14–0.62, *p* = 0.001), and they lived in their own house (OR = 0.49, 95% CI 0.29–0.83; *p* = 0.008).

When the analysis was stratified by the postpartum period, the following findings were obtained. Factors associated with an EPDS > 12 in the first 3 months postpartum (model 2), were younger maternal age (OR = 1.77, 95% CI 1–3.14; *p* = 0.04), the receipt of financial support from the family (OR = 1.56, 95% CI 1.02–2.38; *p* = 0.03), difficulty in managing the monthly income (OR-4.46, 95 CI 1.96–10.1, *p* < 0.001) and a maternity leave of more than 3 months (OR = 4.81, 95%CI 1.52–15.2; *p* = 0.007). From 3 to 6 months postpartum (model 3), not bad management of the monthly income was also a significant risk factor for depressive symptomatology (OR = 1.88, 95% CI 1.04–3.39; *p* = 0.03) in addition to Muslim religion (OR = 4.65, 95% CI 1.07–20; *p* = 0.03) and having a part-time job (OR = 2.94, 95% CI 1.29–6.72; *p* = 0.01). Conversely, having an employed husband (OR = 0.31, 95% CI 0.15–0.64; *p* = 0.001) was found to be protective against postpartum depression in the early postpartum period. In the late postpartum period, women who received the most support from their partner (OR = 0.47, 95% CI 0.29–0.76; *p* = 0.002) and who perceived their infants as healthy were less likely to have an EPDS > 12 (OR = 0.17, 95% CI 0.04–0.65; *p* = 0.01). Similar results were found for women who reported having breastfed their other children (OR = 0.37, 95% CI 0.14–0.97; *p* = 0.04) and had breastfed the past seven days (OR = 0.3, 95% CI 0.16–0.56; *p* < 0.001). Furthermore, living at own home was shown to significantly lower the risk of having an EPDS > 12 in both the early (OR = 0.51, 95% CI 0.28–0.95; *p* = 0.03) and late (OR = 0.34, 95% CI 0.18–0.65; *p* = 0.001) postpartum periods.

## Discussion

The purpose of this study was to investigate the prevalence of postpartum depressive symptomatology and its risk factors among women living in the UAE. Our main findings revealed that 35% of women experienced depressive symptomatology during the first 6 months postpartum. Younger women, Muslim religion, part-time employment, maternity leave more than 3 months, low socioeconomic status, and difficulty in managing family income predicted a higher likelihood of postpartum depressive symptomatology. Breastfeeding, paternal employment status, living in own house, partner support, and perception of a healthy infant predicted a lower likelihood of developing postpartum depressive symptomatology.

The prevalence of postpartum depression in this study is relatively higher than the global pooled prevalence of 17.7% reported by Shorey et al. [3]. Specifically, our results were higher than the figures previously described in the East Asian and western countries in South Korea (1.4%) [19], China (6.7%) [20], Canada (8%) [21], and France (16.7%) [22]. In the UAE, previous studies by Green et al. [11] and Hamdan and Tamim [12] also reported lower rates of depressive symptomatology (22% and 10%, respectively), however, their findings were based on a small sample of only Emirati women [11] and a different screening tool to assess postpartum depression [12]. Comparable results were found in Saudi Arabia (33.2%) [23], Bahrain (37.1%) [24], and recently in the UAE (33%) [7]. These findings are consistent with the meta-analysis by Shorey et al. [3] which found that prevalence estimates were statistically significant when stratified by geographical region (*p* < 0.001), with the Middle East having the highest prevalence of 26% (95% CI 0.13–0.39). The wide range of prevalence rates is due to cross-cultural differences and social diversity [25]. The majority of women in our study were from the Arab region, who perhaps predominantly belong to the collectivistic nature of the Middle Eastern society, where personal problems are overlooked in the interest of the larger family [26]. As a result, women may not practice self-care strategies or seek help and higher rates of postpartum depression symptoms are obtained.

Furthermore, there was a significant decrease in EPDS scores across time. The reduction of depression symptoms in this study may be due to treatment among women who were referred to a clinical psychologist. However, in a Swedish cross-sectional study including 888 mothers, Rosander et al. (2020) reported a peak in the levels of depression symptoms (EPDS ≥ 12) at 9 and 18 months postpartum after observing a comparable decrease in EPDS scores from birth to 6 months postpartum [27]. This suggests the need for a proactive screening program for early identification, management and in addition to follow-up for relapse prevention.

Young maternal age was an important risk factor of postpartum depression in our study and is in line with the available literature where young women between 18 and 25 years old were identified to be at a higher risk of postpartum depression [28, 29]. This could be attributed to the sensitivity of younger women to infant care stress [30], lower self-confidence, and their high help-seeking behavior—established by the high general help-seeking score (GHS) [31] compared to older women. Additionally, Mcmahon et al. [32] revealed that the favorable socioeconomic characteristics of older women such as more financial security, higher educational level, professional occupation, and stability of relationship could be protective against postpartum depression. Similarly, Silverman et al. [30] confirmed the statistically significant association between the risk of postpartum depression and maternal age (*p* < 0.01), however, the authors further declared that this association is modified by the history of depression. Among women with no history of depression, young mothers < 25 years had an increased risk of postpartum depression, whereas mothers with depression history conferred a greater likelihood of postpartum depression in the advanced age ranges 30–39 years [30]. Therefore, results must be interpreted with caution, and further investigation is required to verify the moderating effect of depression history on postpartum depression and maternal age correlation.

In the present study, breastfeeding was a significant protective factor against postpartum depression in the late postpartum period. Substantial research has shown that postpartum depression is highly related to the early discontinuation of breastfeeding. Mothers with early depressive symptoms are more likely to discontinue exclusive or partial breastfeeding and introduce formula feeding [31]. It is hypothesized that postpartum depression impairs the mothers’ self-confidence and interferes with their ability to perform maternal functions, including breastfeeding [32]. Despite this, the correlation between postpartum depression and breastfeeding is also bidirectional. Studies suggested that breastfeeding alleviates depressive symptomatology over time by attenuating the cortisol response to stress [33]. A possible reason could be the skin to skin contact: the longer the duration of skin to skin contact the lower the maternal cortisol levels [33]. Also, maternal self-efficacy and sleep patterns, which are negatively associated with the risk of postpartum depression, seem to be enhanced by breastfeeding [33]. Moreover, mothers who perceived their infants as healthy had a significantly lower risk of postpartum depression. No previous studies have explored and provided a clear understanding of this association. Perhaps maternal self-efficacy is also positively stimulated when the infant is perceived as healthy. Mothers would then experience higher self-esteem in their ability to execute parenting tasks which consequently reduces the risk of postpartum depression [34]. Therefore, mothers should be offered the appropriate pre-and postnatal education to promote awareness of breastfeeding and infant care which in turn could improve their ability in performing their parenting role effectively and competently.

In this study, mothers who had no partner support after childbirth were at a higher risk for developing postpartum depression symptomatology compared to those who had. In a recent systematic review and meta-analysis of 13 studies, women with inadequate social support were 6.27 times more likely to develop postpartum depression compared to those who had adequate social support (POR = 6.27, 95% CI 4.83–8.13, *I*^2^ = 0) [35]. Yamada et al. [27] suggest that several sources of social support may have a differential impact on postpartum depression. Women with no social support from a partner but have social support from others (i.e. parents, relatives, and friends) had a significantly higher risk to develop postpartum depression compared to those who perceived to have both partner and family support (OR = 3.13, 95% CI 2.11–4.63; *p* < 0.05) [27]. Similar to the results of our study, this indicates that the lack of partner support significantly contributes to postpartum depression. Mothers with no partner support should be considered as a high-risk group regardless of the availability of other sources of social support [27].

Maternity leave and employment status are vital aspects of the postpartum period. Our results suggest that maternity leave of more than 3 months is associated with a considerably increased risk of postpartum depression, particularly in the early postpartum period. This is contradictory to previous research which found maternity leave equaling 12 weeks or less increased the risk for postpartum depression [36]. Conversely, employed mothers are more concerned about securing their job after childbirth, and in the context of shorter maternity leave, mothers returning to work earlier may benefit from psychological and social support which bolsters their mental health during the transition into motherhood [36, 37]. Although being a working mother perhaps alleviates the risk of postpartum depression symptoms, it is interesting to note that in our study we found part-time employment predicted a higher risk of postpartum depression from 3 to 6 months postpartum. Ho et al. [38] found comparable results where part-time employment status was a significant risk factor for postpartum depression, which could be related to financial burdens and low income.

Not surprisingly, low income and socioeconomic status are constantly reported as strong risk factors for postpartum depression even in high-income countries [5]. Hamdan and Tamim [12] previously argued that although the average per capita income in the UAE is one of the highest in the world, women in the low socioeconomic status group were more likely to experience postpartum depression compared to those in the higher income group. Women who found it difficult to manage their family income (AOR = 2.37, 95% CI 1.56–3.58; *p* < 0.001) [39] and perceived their economic status as low in comparison to others were more likely to develop postpartum depression symptoms (AOR = 13.33, 95% CI 2.66–66.78; *p* = 0.002) [37]. In addition, the father's employment status was found to be a significant predictor of postpartum depression. This is consistent with the findings of Oztora et al. [26] where husbands of 28.6% of women with probable postpartum depression were unemployed (*p* < 0.05). This demonstrates that partner support could be perceived from different aspects, of which besides childcare, economic support is fundamental [37]. These findings are all in congruence with our study results which suggest that financial security is an important factor that places women at risk for postpartum depression.

With respect to living arrangements, available research has given little attention to its impact on postpartum depression. In this study, mothers who lived in their own home had a statistically significantly lower risk of postpartum depression compared to those who lived in their parents’ or in-law’s house. Vo et al. [40] reported comparable results where women who were living with their parents or in-laws had higher rates of symptoms of postpartum depression (22.0%) compared to those living in their private house (13.8%) (OR = 1.75, 95% CI 1.10—2.80). Green et al. [11] previously explained that with women in the UAE residing in their husbands’ families after marriage, a poor relationship with their mother-in-law may cause marital conflict and thus increase the risk for postpartum depression. However, more research is needed to rationalize the correlation between family living arrangements and postpartum depression.

## Limitations and strengths

Prior history of depression, particularly during the antenatal period, is thoroughly confirmed to be a strong predictor of postpartum depression [41]. However, one of the main study limitations, is that depression was not clinically evaluated during pregnancy. This may have led to an overestimation of the prevalence of postpartum depression. Another limitation is the use of EPDS which is a screening rather than a diagnostic tool, commonly used to identify symptoms of postpartum depression. Also, the sample size is relatively small (*N* = 457) which may restricts the generalizability of the findings to the UAE population of interest. Despite the limitations, the study has multiple strengths. To the best of our knowledge, this is the first cohort study to collect data from various Emirates of the UAE. It included women from different nationalities and ethnic groups which provides a clear representation of mothers residing in the country. The study design is another strength to underscore. A longitudinal cohort study where data were collected prospectively, allows to identify of changes over time and provides insight into cause-and-effect relationships. Although the used Edinburgh Postnatal Depression Scale is a self-report scale, it is widely established to be a valid, reliable, and culturally appropriate screening tool to detect probable postpartum depression. Finally, numerous potential covariates from prior literature were accounted for in the present study.

## Implications and conclusions

This study provides evidence that a considerable proportion of women living in the UAE experience postpartum depression symptomatology. Younger age, part-time employment, low socioeconomic status, maternity leave of more than 3 months, and difficulty in managing family income were found to be significant risk factors whereas breastfeeding, partner employment status, living in own house, partner support, and the perception of a healthy infant, were found to have a protective effect. Our findings suggest the importance of developing an innovative multidisciplinary approach to managing perinatal mental health. First, healthcare providers should receive the appropriate training on postpartum depression. To be aware of the significant risk factors prevailing among women living in the UAE, to conduct early screening for postpartum depression, to provide timely therapeutic interventions, and finally to routinely follow-up for relapse prevention. Second, as part of the perinatal care, all women should be educated about the challenges and coping strategies during the postpartum period along with the postpartum depression symptoms to promote self-referral when needed. Also, there’s a strong need for a family-level intervention involving the provision of sufficient father support for the mother and positive co-parenting behaviors between the parents to prevent the development of postpartum depression and encourage early help-seeking when needed. Further research is pivotal to confirm the associated risk factors of postpartum depression among women in the UAE in the early versus late postpartum period. In addition, to implement an evidence-based standardized system, including a structured screening protocol, to optimize postpartum care in the UAE.
